# Curved Surfaces Induce Metachronal Motion of Microscopic Magnetic Cilia

**DOI:** 10.1021/acsami.4c06884

**Published:** 2024-07-10

**Authors:** Zhiwei Cui, Tanveer ul Islam, Ye Wang, Jaap M. J. den Toonder

**Affiliations:** † Microsystems, Department of Mechanical Engineering, Eindhoven University of Technology, 5612 AE Eindhoven, The Netherlands; ‡ Institute for Complex Molecular Systems (ICMS), Eindhoven University of Technology, 5612 AJ Eindhoven, The Netherlands

**Keywords:** metachronal motion, magnetic artificial cilia, miniaturization, flow pattern transportation, particle transportation

## Abstract

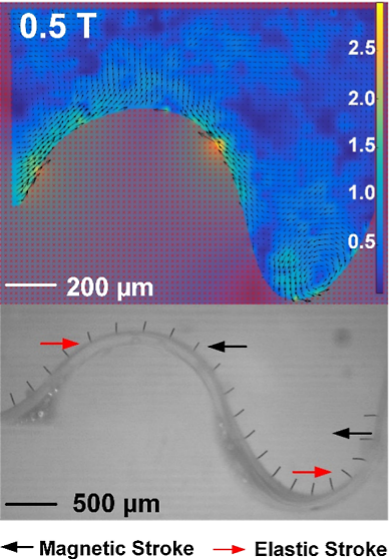

Cilia are hair-like organelles present on cell surfaces. They often exhibit a collective wave-like motion that can enhance fluid or particle transportation function, known as metachronal motion. Inspired by nature, researchers have developed artificial cilia capable of inducing metachronal motion, especially magnetic actuation. However, current methods remain intricate, requiring either control of the magnetic or geometrical properties of individual cilia or the generation of a complex magnetic field. In this paper, we present a novel elegant method that eliminates these complexities and induces metachronal motion of arrays of identical microscopic magnetic artificial cilia by applying a simple rotating uniform magnetic field. The key idea of our method is to place arrays of cilia on surfaces with a specially designed curvature. This results in consecutive cilia experiencing different magnetic field directions at each point in time, inducing a phase lag in their motion, thereby causing collective wave-like motion. Moreover, by tuning the surface curvature profile, we can achieve diverse metachronal patterns analogous to symplectic and antiplectic metachronal motion observed in nature, and we can even devise novel combinations thereof. Furthermore, we characterize the local flow patterns generated by the motion of the cilia, revealing the formation of vortical patterns. Our novel approach simplifies the realization of miniaturized metachronal motion in microfluidic systems and opens the possibility of controlling flow pattern generation and transportation, opening avenues for applications such as lab-on-a-chip technologies, organ-on-a-chip platforms, and microscopic object propulsion.

## 1 Introduction

Cilia, slender hair-like external cell organelles with a length between 1 and 30 μm, are ubiquitously present in numerous organisms in the natural world.^1−3^ These tiny structures play a pivotal role in various biological processes, from the propulsion of microscopic Paramecium, to the clearing of the human respiratory tract, and to the transport of the ovum in fallopian tubes. Individual cilia exhibit asymmetric beating composed of an effective stroke, in which the cilium beats straighter, and a recovery stroke, in which the cilium undergoes a more curved shape moving more closely to the surface.^4−6^ A fascinating phenomenon in the collective behavior of cilia often observed in nature is metachrony, in which neighboring cilia move slightly out of phase, resulting in a wave-like overall motion.^7−10^ Nature showcases four types of metachronal motions categorized by the wave propagation directions in relation to the beating motion of individual cilia: symplectic, antiplectic, dexioplectic, and laeoplectic motion, where the wave propagates in the same direction, opposite to, perpendicularly leftward to, and perpendicularly rightward to the cilium effective stroke, respectively.^1,7^ This metachronal motion helps to enhance the cilium function, for example, mucus clearance in the human respiration system,^10−14^ ovum transportation in fallopian tubes,^15^ and propulsion for organisms like Paramecia.^8,9,16^ Some organisms, like the starfish larvae, can dynamically change their cilium beating pattern, effectively controlling fluid vortices around them, which provides a unique advantage in navigating complex environments.^17^

Motivated by the prominence of metachronal motion of cilia in various biological processes, researchers have engineered artificial cilia capable of metachronal motion driven by diverse stimuli, including electric field,^18−20^ pneumatics,^21−24^ light,^25−28^ and magnetic field.^5,6,17,29−37^ Among these, magnetically responsive artificial cilia stand out for exhibiting a rapid and reversible response and can be actuated remotely without the need for intricate external physical connections. Moreover, magnetic fields can effectively and harmlessly penetrate biological tissues.^5,27^ Because of these advantages, the possibility of inducing metachronal motion by magnetic artificial cilia has been increasingly investigated recently. Two primary approaches have been explored to induce phase differences in a beating magnetic artificial cilium array. The first approach involves applying a periodic nonuniform magnetic field to an array of identical cilia, for example, through a setup comprising rod-shaped magnets with opposing dipole orientations, arranged on a translating belt.^6^ This generates a time-dependent nonuniform magnetic field, creating phase differences between neighboring cilia. The second approach modifies the magnetic properties of consecutive cilia by altering the magnetic particle distribution in neighboring cilia or by changing the length of the cilia within an array.^5,17,29,31,36,37^ However, both approaches are facing complexities and size limitations, which make it difficult to apply them at small scales. The first approach is limited by the size of the actuating magnets, and the second method is limited by the complexity of fabricating small cilia with individual control of shape or magnetic particle distribution. Recently, a third method has been introduced by our group, integrating a paramagnetic substructure beneath an array of identical magnetic artificial cilia and actuating by a uniform magnetic field.^35^ Due to locally generated perturbations of the magnetic field, a time-dependent periodic nonuniform magnetic field induces metachronal motion of the cilia. This method overcomes the size limitation, achieving the miniaturization of metachronal motion. Nonetheless, the fabrication process remains complex, and the integration of cilia with the substructure is intricate.

Addressing these challenges, we introduce a completely new, fourth approach to achieve the metachronal motion of magnetic artificial cilia. This method eliminates the need for individual cilium property control or complex magnetic field generation, offering a remarkably straightforward solution to induce metachrony. The key idea is to attach an array of identical magnetic cilium arrays on a curved surface, as opposed to a flat one, and to actuate them using a simple rotational uniform magnetic field. The surface curvature induces varying magnetic torques on neighboring cilia due to the differences in the angles between the cilium axes and the applied magnetic field. This innovative technique enables one to design metachronal patterns simply by modulating surface curvature, mimicking symplectic and antiplectic metachronal motion of biological cilia. Moreover, both metachronal motions can be combined in one cilium array simultaneously by specifically designing the surface profile. We note that our curved surface is reminiscent of some ciliated surfaces, for instance, the inner surfaces of trachea.^38^ The curved surface not only mirrors natural environments but also leads to specific local fluid flow, as revealed by our flow characterization experiments, which show the emergence and transportation of vortical patterns.

Our method may offer a new approach to enable diverse applications, for example, inducing flow patterns in microfluidic devices with applications in lab-on-a-chip and organ-on-a-chip or realizing propulsion of microscopic objects. Furthermore, it may provide the future possibility of investigating and understanding the fluid dynamics in biological environments, which potentially enriches our understanding of the functioning of complex biological systems, such as the human respiratory tract.

## 2 Results and Discussion

### 2.1 Magnetic Artificial Cilium Fabrication and Actuation

**Figure 1:**
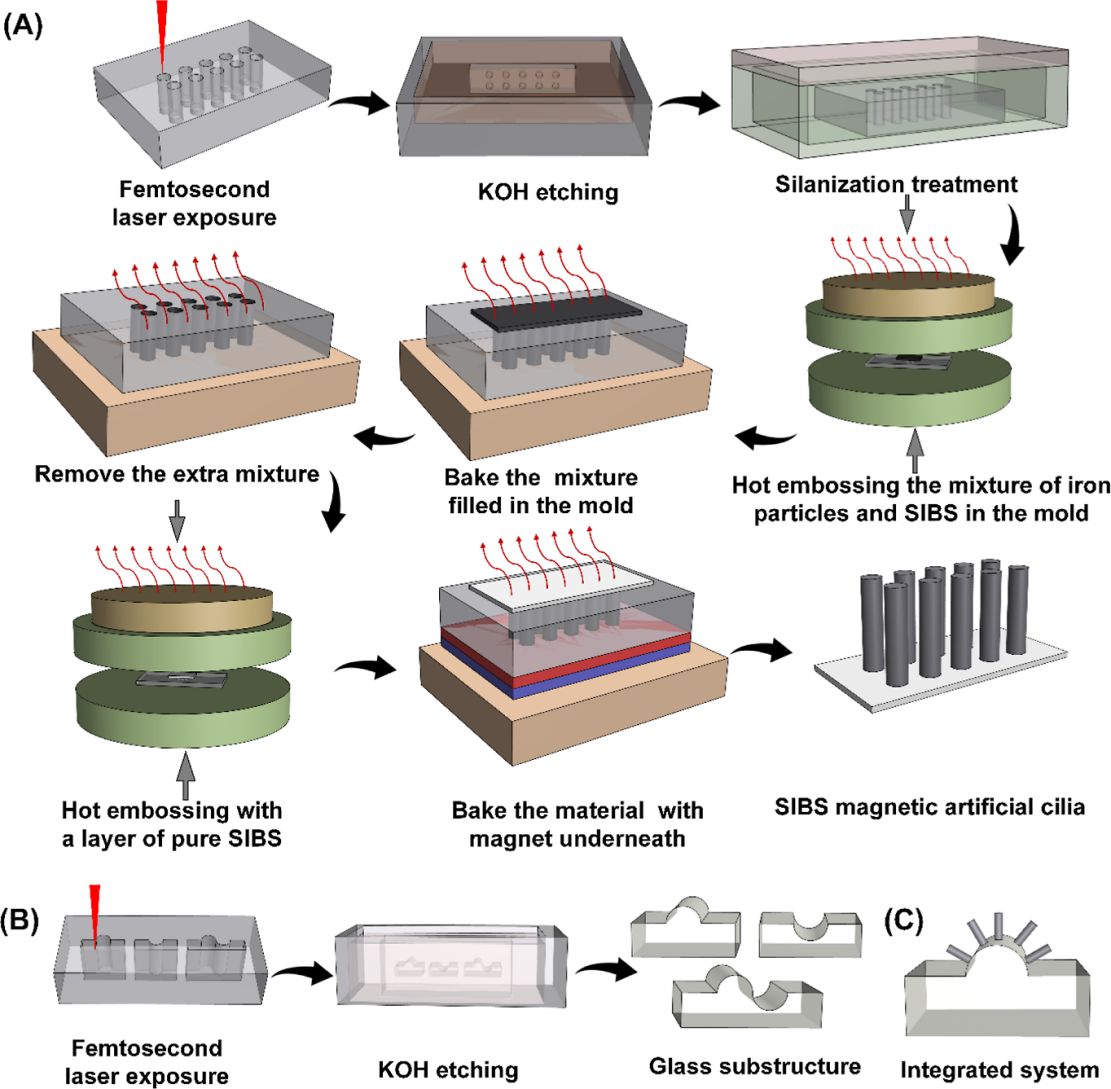
Fabrication process of microscopic magnetic artificial cilia and glass surfaces. (A) Schematic of the fabrication process of the magnetic artificial cilia. We used femtosecond laser machining to fabricate the mold and applied the hot embossing method to fill the mold. (B) Schematic of the fabrication process of the glass surfaces. We fabricated three types of surface geometries as illustrated here. (C) Schematic of the integrated magnetic artificial cilia and curved glass surface.

**Figure 2:**
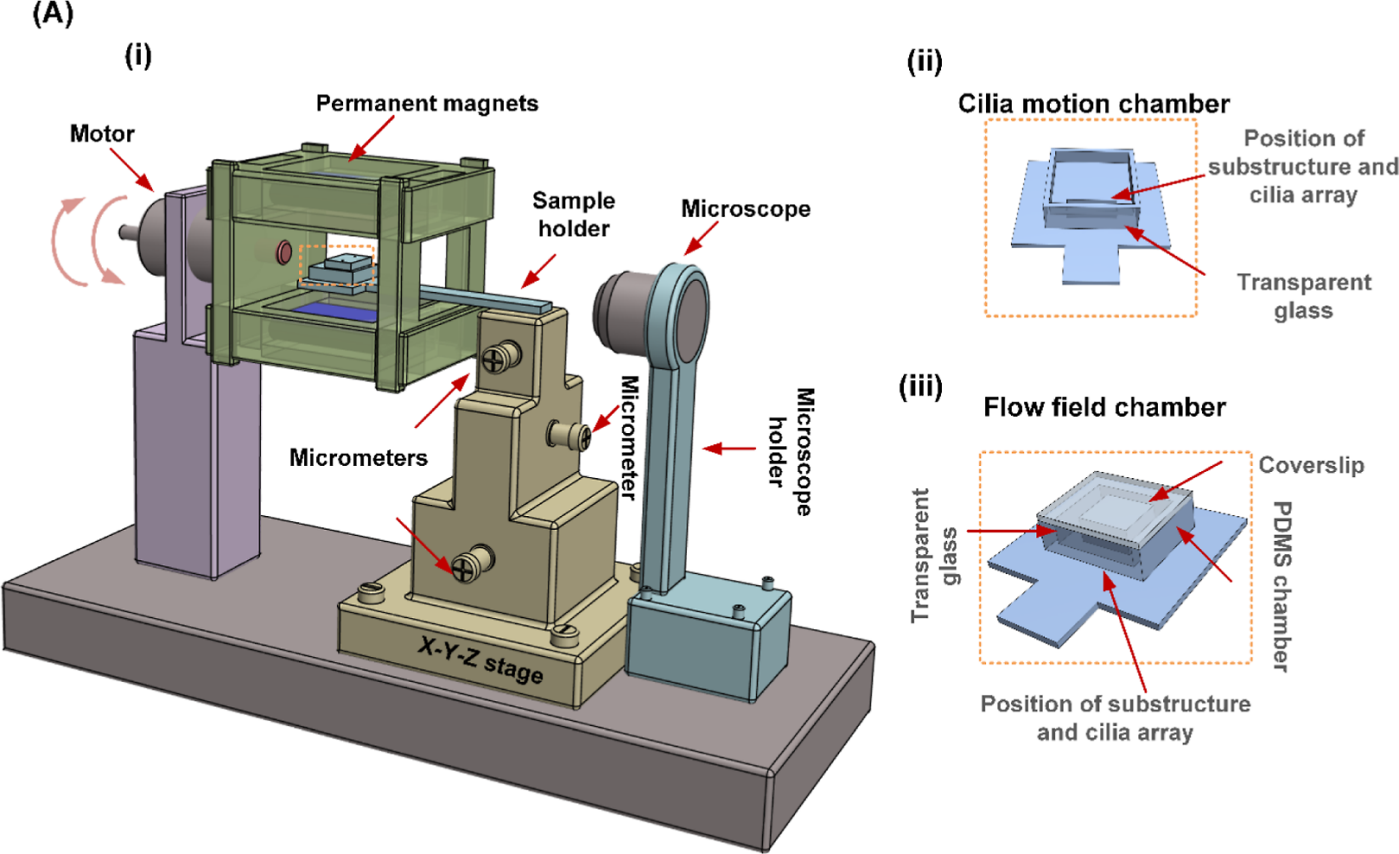
Actuation setup and holders used to actuate the cilia and observe the magnetic cilium motion and flow patterns. (i) Schematic of the home-built magnetic actuation setup which can induce a rotating uniform magnetic field of around 150 mT at the center of the working space. (ii) Schematic of the 3D-printed chamber allowing for observing the cilium motion. (iii) Schematic of the closed PDMS chambers for observing the flow patterns.

We fabricated microscopic magnetic artificial cilia, with a diameter of 15 μm, a length of 150 μm, and a center-to-center distance of 350 μm, by applying a micromolding process as shown in Figure 1A. Details are provided in the materials and methods section. The cilium array was constructed using styrene-isobutylene-styrene triblock copolymer (SIBS) polymer and paramagnetic carbonyl iron powder. SIBS was selected due to its favorable characteristics for our specific application: the commendable tensile properties of the polymer ensured a high demolding rate even for the substantial aspect ratio (10) of the cilia with relatively small dimensions; also, the relatively low elastic modulus of SIBS made them deflect significantly under a magnetic torque. Note that we baked the material before demolding at 160 °C in the presence of a magnet to achieve an identical distribution of the magnetic particles in each cilium in the array. A curved glass surface was crafted by direct writing on a fused silica slide using femtosecond laser machining followed by wet etching to achieve the intended design as illustrated in Figure 1B. To induce metachronal motions characterized by distinct wave propagations, we engineered three variants of a glass surface featuring semicylindrical shapes: the first with a convex surface with a diameter of 1 mm, the second presenting a concave surface with a diameter of 2 mm, and the third possessing both convex and concave surfaces, with a diameter of 2 mm, as depicted in Figure 1B. Subsequently, we attached the fabricated cilium patch on top of the glass surfaces, a side view of which is depicted in Figure 1C. Then we mounted the integrated cilium system onto a holder and actuated it with a counterclockwise rotational uniform magnetic field with a magnitude of 150 mT in the central area, shown in Figure 2A(i). Detailed information about the actuation setup is available in the materials and methods section.

### 2.2 Surface Curvature Induces Metachronal Motion of Microscopic Magnetic Artificial Cilia

**Figure 3:**
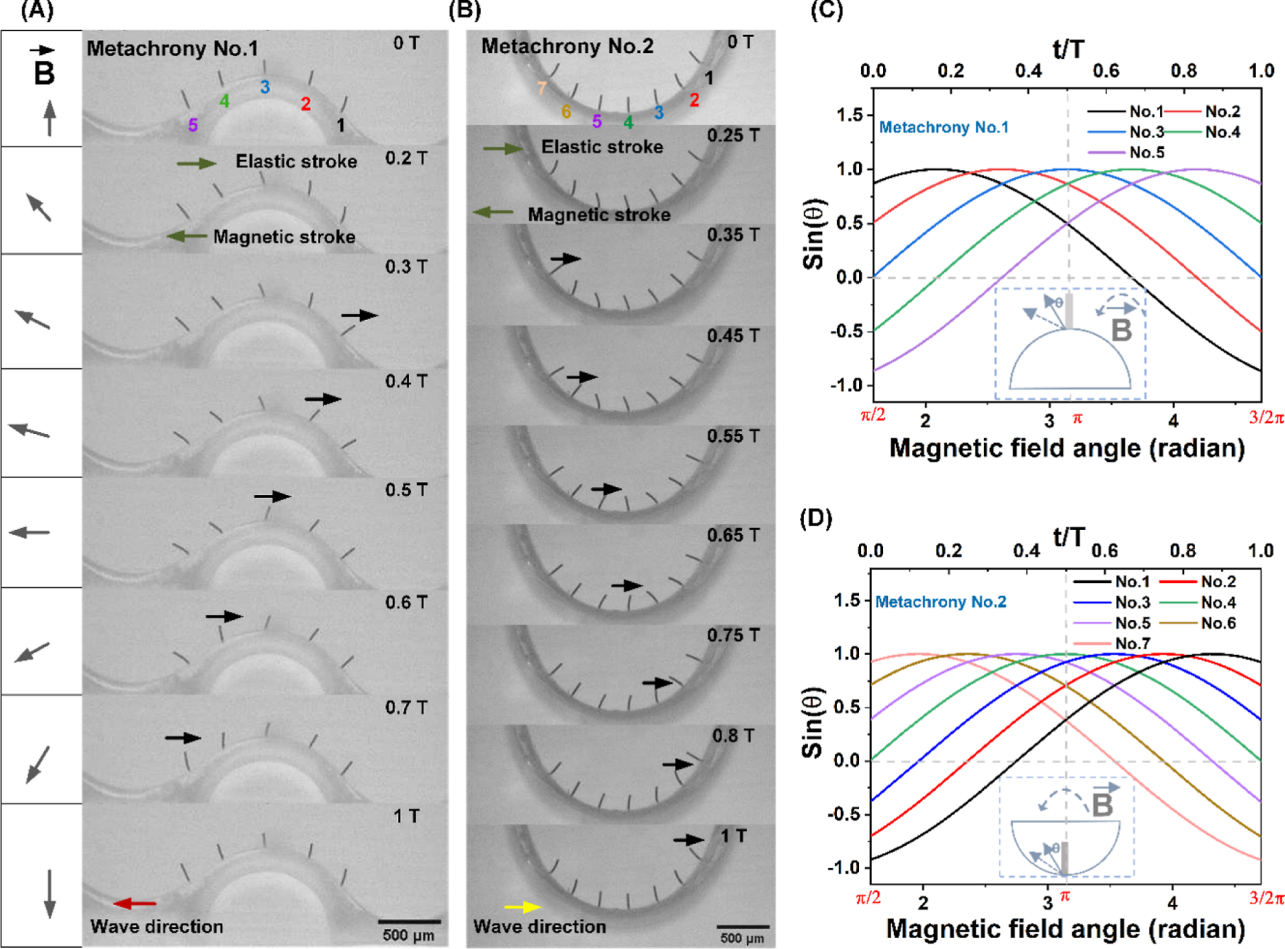
Two types of metachronal motion of magnetic artificial cilia induced by surface curvature. (A) Side view of the metachronal motion of magnetic artificial cilia: metachrony no. 1. The cilium array is attached to a glass surface with a convex surface with a radius of curvature of 0.5 mm, and the cilia are actuated by a counterclockwise rotating uniform magnetic field with a magnitude of around 150 mT. The black arrows indicate cilia making their elastic stroke. The metachronal wave propagates to the left. The corresponding video is available in Movie S1. The cilium beating frequency is 1 Hz. (B) Side view of the metachronal motion of magnetic artificial cilia: metachrony no. 2. Similarly, the cilium array is attached to a glass substructure with a concave surface with a radius of curvature of 1 mm, and the cilia are actuated by a counterclockwise rotating uniform magnetic field with a magnitude of around 150 mT. The black arrows indicate cilia making their elastic stroke. The corresponding video is available in Movie S2. The cilium beating frequency is 1 Hz. (C) The change of sin(θ) for all five cilia during the rotation of the magnetic field in the case of metachrony no. 1. θ is defined as the angle between the initial direction of the nonactuated cilia and the direction of the magnetic field, as shown in the inset for cilium 3. (D) The change of sin(θ) for all seven cilia during the rotation of the magnetic field in the case of metachrony no. 2. θ is defined as the angle between the initial direction of the nonactuated cilia and the direction of the magnetic field, as shown in the inset for cilium 4.

To facilitate a clear observation of the motion of the cilium array, we printed a chamber with a glass slide glued in front, as shown in Figure 2A(ii). The integrated cilium system was positioned within the recess of the chamber, allowing for a further examination of cilium motion in water. The resulting motion from actuating with a counterclockwise rotating magnetic field is shown in Figure 3A,B in side view; the corresponding videos are available in Movies S1 and S2. Clearly, neighboring cilia present a phase difference resulting in metachronal motion, called “metachrony no. 1” and “metachrony no. 2″, respectively, since the metachronal wave propagates in opposite directions. In both metachronal motions, each individual cilium exhibits 2D reciprocal motion that includes a magnetic stroke and an elastic stroke, which are indicated in the figures. This is similar to the motion observed in refs (5),^6^, and^37^. Specifically, the magnetic field commences from an initial orientation of 90° and undergoes subsequent counterclockwise rotation as indicated in Figure 3A. An individual cilium, initially aligned with the magnetic field (e.g., cilium 3 in Figure 3A at time zero), follows the rotating motion of the magnetic field initially due to the magnetic torque acting on it, which leads to the cilium bending leftward; this phase is called “magnetic stroke”. During bending, the cilium accumulates elastic energy. At some point, the magnetic energy cannot compensate for the accumulated elastic energy, and as a consequence, the cilium whips back, which is called the “elastic stroke”. The cilium tip whips back beyond its initial position, followed by the reengagement by the magnetic field, initiating a subsequent leftward bending motion to its starting position, and the next magnetic stroke starts. This repeating cycle has a duration T. The cilia do not show any obvious shape asymmetry, i.e., the magnetic and the elastic strokes follow the same path and the net swept area is zero. Note that the cilium beating frequency is 2 times the rotating frequency of the magnetic field due to the symmetry of the generated magnetic field in the first and the second halves of one rotating cycle of the field. Even though the directions of the magnetic fields are opposite to each other in the two different cycle halves, this has no effect on the bending behavior of the cilia.

The resulting metachronal cilium motion of metachrony no. 1 shown in Figure 3A can be understood from an analysis of the time evolution of the local magnetic field direction relative to the initial, nonactuated orientation of each cilium, represented by the angle θ in Figure 3C. The numerical labels and colors in Figure 3A correspond to those in Figure 3C, which shows sin(θ) for all five cilia, chosen since the magnetic torque is proportional to the sine of the angle between the magnetic moment of the cilia and the magnetic field.^39,40^ Focusing on cilium no. 3 first, it is initially aligned with the magnetic field at time *t* = 0 T, when the magnetic field angle is 90° (or π/2), and hence its θ is zero and sin(θ) is zero as well. Subsequentially, as the magnetic field rotates, θ increases and hence sin(θ) increases, and cilium 3 performs its magnetic stroke. When the magnetic field reaches 180° (or π) at *t* = 0.5 T, the θ of cilium no. 3 is 90° and sin(θ) = 1 is maximal and then starts decreasing. At this stage, the cilium initiates the whipping back process, occurring just after *t* = 0.5 T for cilium no. 3, as seen in Figure 3A. After moving beyond its initial position, cilium no. 3 is recaptured by the external magnetic field, starting its magnetic stroke again bending leftward. Hence, the cilium commences its elastic stroke near the peak values of its sin(θ), corresponding to an angle θ of approximately 90°. The initial, nonactuated orientations of cilium nos. 1, 2, 4, and 5 are shifted by −60, −30, 30, and 60°, respectively, as can be seen from Figure 3A, and hence the sine curves in Figure 3C are shifted by the same angles. This explains the corresponding phase shift of the elastic stroke and the resulting metachrony shown in Figure 3A.

As previously mentioned, nature presents various types of metachronies determined by the direction of the metachronal wave propagation with respect to the direction of the effective stroke of the cilia, particularly symplectic and antiplectic metachrony, in which the metachronal wave propagates in the same and in the opposite direction of the effective stroke, respectively. In artificial cilia, the metachronal wave propagation and the cilium stroke asymmetry can mostly not be controlled separately. This difficulty arises from the fact that reversing the metachronal wave propagation, for example, by reversing the rotation of the magnetic field, also results in the reversal of individual cilium motions, causing the entire motion to mirror completely.

With our novel method, we can realize different types of metachronies, analogous to symplectic and antiplectic metachrony in nature, simply by changing the curvature of the surface, as shown in Figure 3B. For “metachrony no. 2” shown here, the individual cilium motion remains identical to that in metachrony no. 1 shown in Figure 3A, with a magnetic stroke to the left and an elastic stroke to the right, when the external magnetic field undergoes counterclockwise rotation. However, the wave propagation direction of metachrony no. 2 is to the right, opposite metachrony no. 1, as is evident from Figure 3B. This behavior can be explained by an analysis of the development of the angle θ between the initial, nonactuated cilium orientation and the magnetic field, the sine of which is shown in Figure 3D for all cilia in the array. Each individual cilium undertakes a magnetic stroke and an elastic stroke, with the elastic stroke of a cilium initiating at approximately the peak value of its sine curve, just like in metachrony no. 1. However, the order in which the cilia reach the peak value in metachrony no. 2 is reversed compared to metachrony no. 1, due to the inverted curvature of the surface, which mirrors the phase angles between the initial θ of the cilia. Hence, in metachrony no. 2, the wave starts from cilium no. 7 and propagates to no. 1, as seen in Figure 3B, consistent with the timing of the sine peaks in Figure 3B.

### 2.3 Surface Curvature Design Enables the Integration of Metachrony No. 1 and No. 2 in One Cilium Array

To demonstrate the versatility of using curved surfaces to achieve different types of metachronal motions, we designed a cilium array capable of simultaneously exhibiting both symplectic and antiplectic metachronal motion, effectively integrating metachrony no. 1 and no. 2. This was accomplished by designing and fabricating a surface featuring both concave and convex curvatures, as illustrated in Figure 1B.

**Figure 4:**
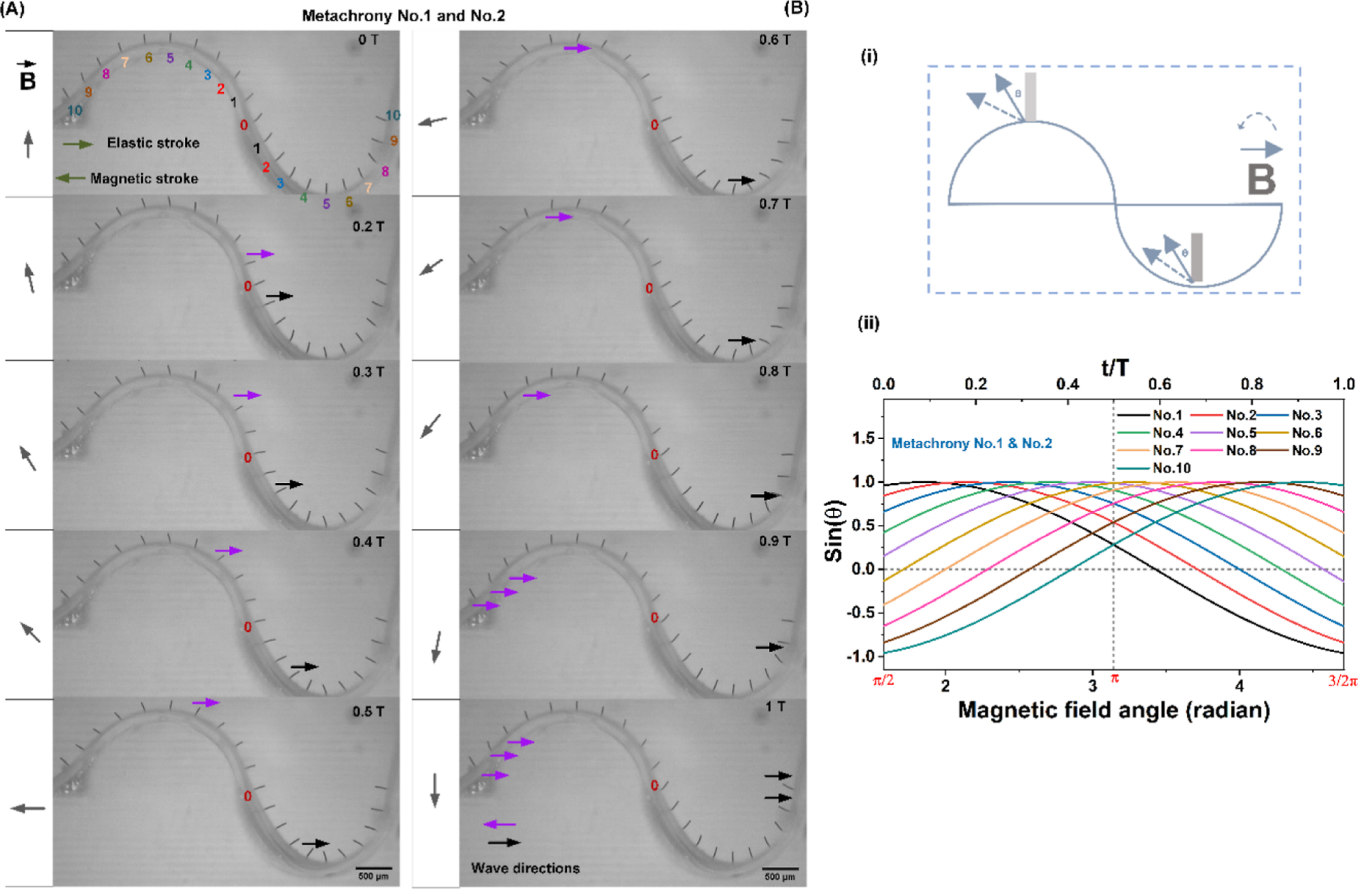
Demonstration of different metachrony types combined on a single surface. (A) Side view of the metachronal motion of magnetic artificial cilia: combination of metachrony no. 1 and no. 2. The cilium array was attached to a glass surface having a combined convex–concave structure with the radii’s curvature of 1 mm, and the cilia were actuated by a counterclockwise rotating uniform magnetic field of around 150 mT. The purple arrows indicate cilia on the convex surface undergoing their elastic stroke, while the black arrows point to cilia on the concave surface undergoing their elastic stroke. A corresponding video is available in Movie S3. The cilium beating frequency is 1 Hz. (B) (i) Definition of θ, the angle between the initial direction of the nonactuated cilia and the direction of the magnetic field; (ii) the change of sin(θ) for all cilia shown in panel (A) during the rotation of the magnetic field.

The resulting motion is shown in Figure 4A in a side view. A corresponding video is available in Movie S3. The label numbers and colors in Figure 4A correspond to those in Figure 4B(ii) which shows the evolution of the sine of the angle θ between the initial direction of the nonactuated cilia and the direction of the magnetic field. Notably, the label numbers for the concave structure are arranged from left to right, mirroring the arrangement in the convex structure. The purple arrows in Figure 4A indicate cilia on the convex structure making an elastic stroke; the black arrows point to cilia in the concave structure making an elastic stroke.

In Figure 4A, the metachronal wave propagates to the left on the convex segment, exhibiting metachrony no. 1 consistent with Figure 3A. Conversely, the metachronal wave travels to the right on the concave segment, showing metachrony no. 2 consistent with Figure 3B. The observed behavior and timing of the elastic strokes seen in Figure 4A is again in agreement with the evolution of sin(θ), like in Figure 3: cilia undergo an elastic stroke when the corresponding sin(θ) is approximately equal to 1, see Figure 4B(ii). Exceptions are cilium nos. 8 to 10 on the convex segments which exhibit almost synchronous backward movement, as can be seen in Figure 4A and Movie S3. This behavior can be attributed to local inaccuracies in the attachment of the cilium patch to the curved surface, resulting from the gluing process that introduced a thin layer of glue beneath the entire cilium array but the accumulation of excess glue at some locations hindered the cilium array from precisely conforming to the surface curvature. A similar phenomenon was observed on the concave segment for cilium nos. 9 and 10. This practical issue can be resolved by using a more accurate attachment procedure.

Figure 4 demonstrates that the metachrony can be controlled by designing surface curvature, providing great flexibility to attain the desired metachronies across various segments of the same or different surfaces. The metachrony can be further controlled through changing parameters such as surface curvature dimensions, number of cilia, and cilia–cilia distance.

### 2.4 Surface Curvature-Induced Cilium Metachrony Generates Diverse Flow Patterns

Generating flow is an important application of artificial cilia, and the flow induced by metachronal artificial cilia realized in various ways has been investigated through both numerical simulations and experimental investigations.^6,8,17,37^ Since metachrony in biological systems typically occurs at low Reynolds numbers, we conducted a study of the flow patterns generated by the cilium metachrony induced by curved surfaces in a highly viscous liquid, specifically glycerol, thus simulating a low-Reynolds-number condition.

**Figure 5:**
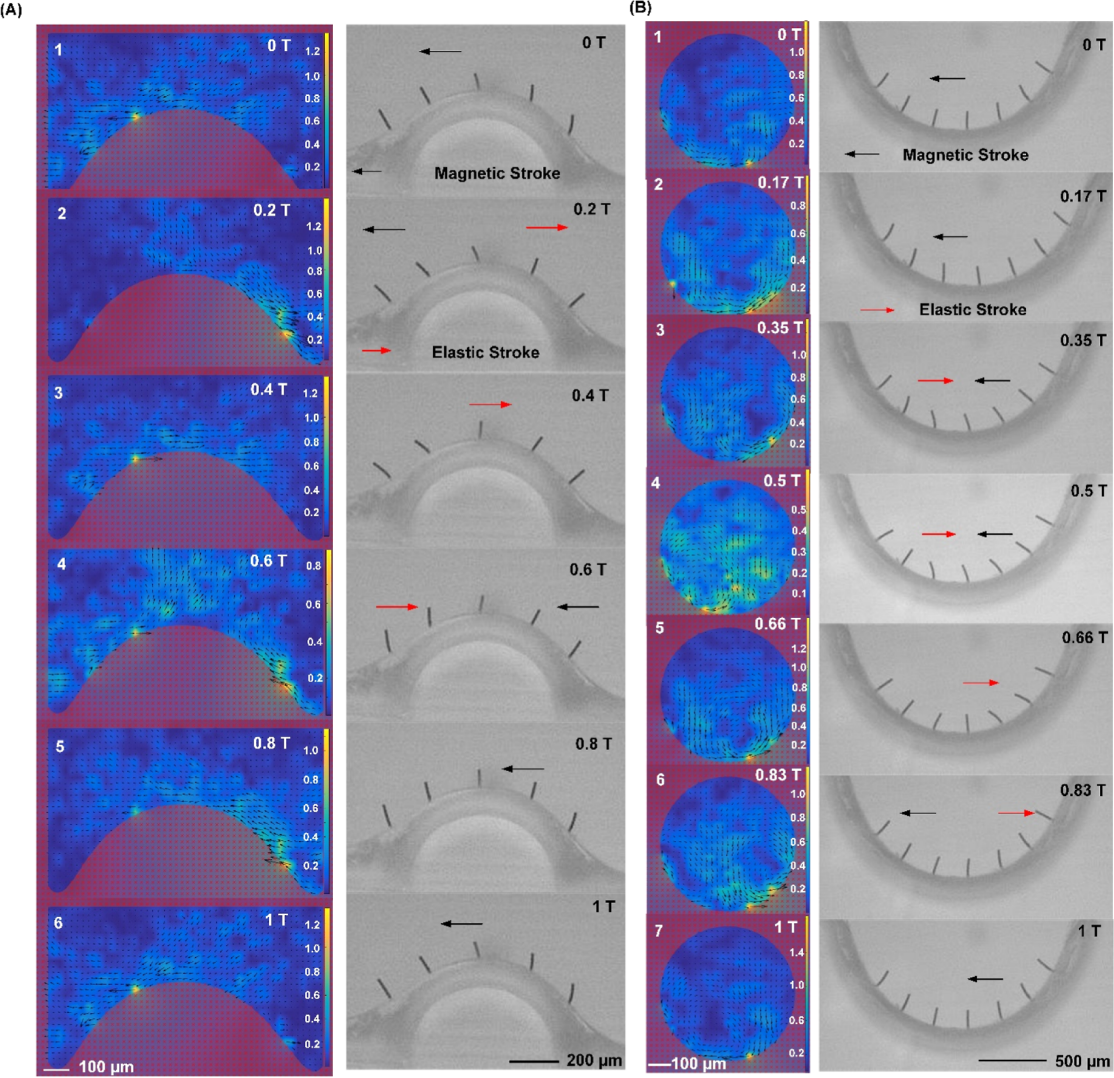
Local flow pattern generated by metachrony no. 1 and metachrony no. 2 and the corresponding cilium motion. (A) The left row shows snapshots of the fluid velocity distribution at 6 time points in one actuation cycle. The black arrows indicate the flow direction, and the length of the arrows together with the color bars indicates the magnitude of the velocity. The unit of the color bars is μm/s. The right row shows the corresponding cilium motion at the same time points. The black arrows indicate that the cilia undergo their magnetic strokes, while the red arrows point to the cilia that undergo their elastic stroke. The cilium beating frequency is 5 Hz. (B) The left row shows snapshots of the fluid velocity distribution at 7 time points in one actuation cycle. The black arrows indicate the flow direction, and the length of the arrow together with the color bars indicates the magnitude of the velocity. The unit of the color bars is μm/s. The right row shows the corresponding cilium motion at the same time points. The black arrows indicate that the cilia undergo their magnetic strokes, while the red arrows point to the cilia that undergo their elastic stroke. The cilium beating frequency is 5 Hz.

The local flow pattern generated by metachrony no. 1, on the convex surface, is shown in Figure 5A. The top section of the figure shows the resulting flow patterns at different time points in one actuation cycle, while the bottom section shows the corresponding cilium motion. Note that the starting point of this sequence (i.e., *t* = 0) is not exactly equal to that of Figure 3A. As depicted in Figure 5A, initially, all cilia in the patch exhibit leftward movement (*t* = 0 T), which means they are all in their magnetic stroke phase as indicated by the black arrow in the figure. Consequently, the resultant flow is directed to the left, as illustrated in Figure 5A(1). With the rotation of the magnetic field, the cilia start making their elastic strokes sequentially, moving rightward when the accumulated elastic energy surpasses the magnetic energy. Consistent with Figure 3A, the cilium array exhibits metachronal motion with the wave propagating leftward. In Figure 5A(2) (*t* = 0.2 T), the rightmost cilium has just finalized its elastic stroke and the cilium next to it is undergoing its elastic stroke, leading to the observed local rightward flow. Since the whipping motion during the elastic stroke is faster than the more gradual bending during the magnetic stroke, the generated local velocity shows a temporary peak. At *t* = 0.6 T, a local flow to the left is again observed on the right side of the convex structure, as the cilia there have resumed their magnetic strokes after completing their elastic strokes, but on the left side of the convex structure, the flow is to the right because the leftmost cilia are just finalizing their elastic stroke. Hence, for metachrony no. 1, we see a general flow generation to the left due to the magnetic cilium strokes, with a local peak of rightward flow generation that propagates to the left, in the metachronal wave direction, due to the sequential elastic strokes.

The local flow pattern induced by metachrony no. 2, on the concave surface, is shown in Figure 5B. Note that the starting point of this sequence (*t* = 0) is not the same as that in Figure 3B. It can be seen that all the cilia are in their magnetic stroke at the starting point of the cycle (*t* = 0 T), moving leftward, which results in a general local flow to the left. As the magnetic field rotates, the cilia bend further, intensifying the flow while still remaining in their magnetic strokes, as indicated in Figure 5B(2). The concave curvature of the surface confines the flow, in contrast to the convex surface of Figure 5A, prompting vortex formation with counterclockwise rotation, which is clearly observed in Figure 5B(2). Subsequently, the cilium on the left initiates its rightward elastic stroke, around *t* = 0.35 T, leading to a local rightward flow. Because of the concave curvature, the local flow due to the elastic stroke gives rise to the generation of a second vortex with clockwise rotation, as clearly seen in Figure 5B(3). As the cilia sequentially commence their elastic strokes, this local vortex is transported from left to right, following the metachronal wave propagation, as shown in Figure 5B(4–6). Eventually, the cilia return to their initial positions and repeat the cycle again, as shown in Figure 5B(7). Hence, for metachrony no. 2, we see the appearance of a general counterclockwise vortex in the concave structure generated by the magnetic cilium strokes, with a superposed smaller clockwise vortex propagating to the right, in the metachronal wave direction, due to the sequential elastic strokes.

**Figure 6:**
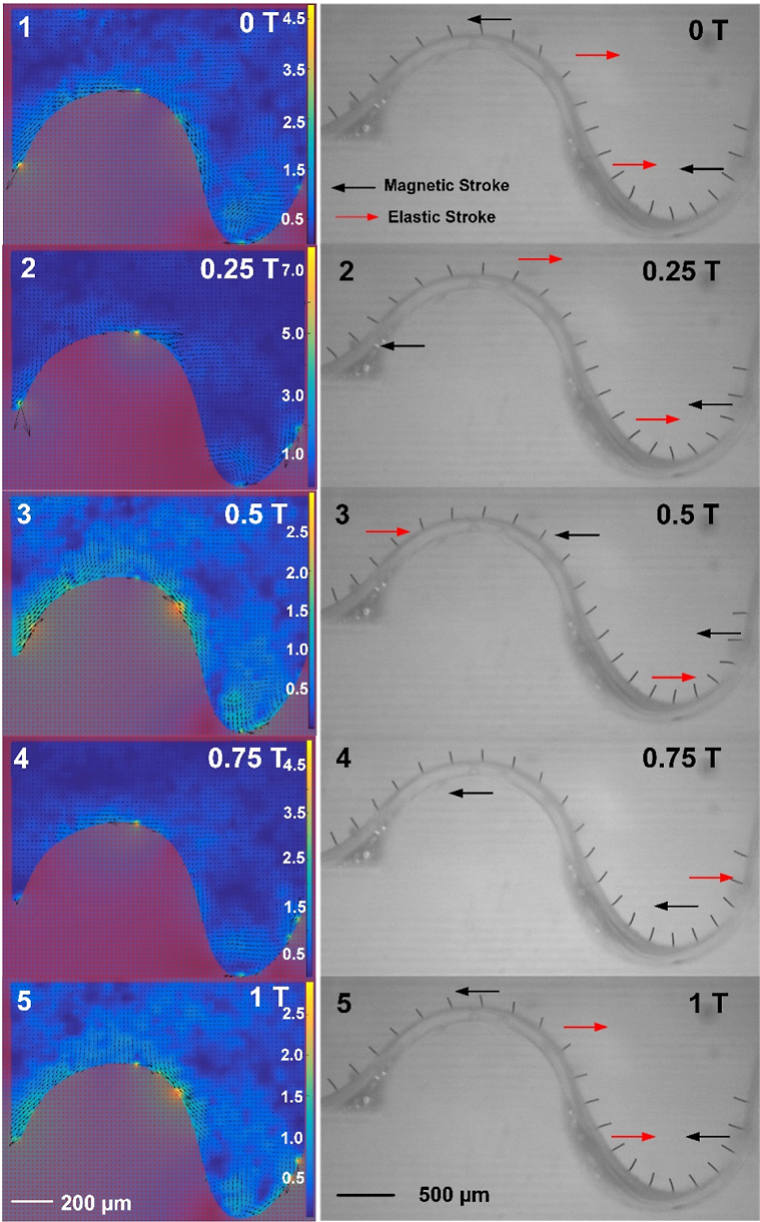
Local flow pattern generated by the cilium array exhibiting combined no. 1 and no. 2 metachrony, and the corresponding cilium motion. The left row shows snapshots of the fluid velocity distribution at 5 time points in one actuation cycle. The black arrows indicate the flow direction, and the length of the arrow together with the color bars indicates the magnitude of the velocity. The unit of the color bars is μm/s. The right row shows the corresponding cilium motion at the same time points. The black arrows indicate that the cilia undergo their magnetic strokes, while the red arrows point to the cilia that undergo their elastic stroke. The cilium beating frequency is 5 Hz.

The flow pattern resulting from the combined presence of both metachrony no. 1 and no. 2 is shown in Figure 6. Again, the starting point (*t* = 0) of this sequence is different from that in Figure 4A. Generally, the flow patterns observed in Figure 6 on the convex and concave parts are consistent with those observed in Figure 5A,B, respectively. On the convex structure, the magnetic strokes generate a general leftward flow, and the sequential elastic strokes induce a temporary local rightward flow that propagates to the left. In the concave structure, there is a global counterclockwise vortex that is generated by the magnetic strokes and a local clockwise vortex propagating to the right due to the sequential elastic strokes. Due to the interreference between the two regions, however, the flow patterns are not as pronounced as for the individual convex and concave structures.

**Figure 7:**
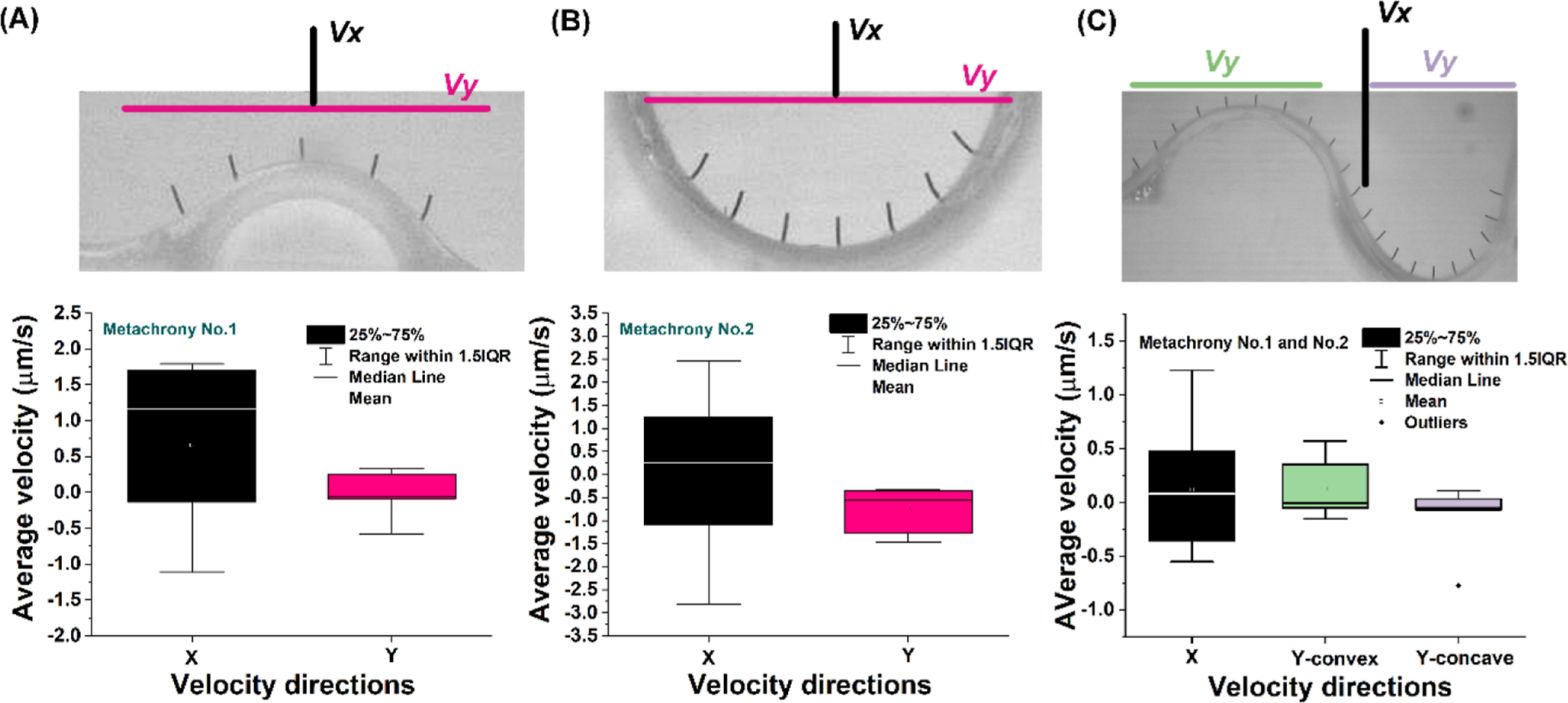
Net flow velocity generated by the surface-induced cilium metachrony. Net flow velocity generated in both *x* and *y* directions, calculated by averaging the corresponding velocity components over the black line section (for the *x* component) and over the pink line section (for the *y* component), as well as over multiple cycles, for metachrony (A) no. 1 and (B) no. 2. (C) Net flow velocity generated by the cilium array exhibiting combined no. 1 and no. 2 metachrony, in both *x* and *y* directions, calculated by averaging the corresponding velocity components over the black line section (for the *x* component), over the green line section (for the *y* component above the convex structure), and over the purple line section (for the *y* component above the concave structure), as well as over multiple cycles.

To investigate whether the metachronal motion generates any net flow, we computed the average flow velocities both in the *x* and the *y* direction by averaging the measured velocities over vertical and horizontal line sections and over multiple cycles, as illustrated in Figure 7. For all cases, and in both directions, there is no significant net flow, considering the extremely low values and the relatively large standard deviation. Based on the low-Reynolds-number conditions and the symmetric motion of the individual cilia, this may have been expected. However, these results indicate that the metachronal motion also does not contribute to net flow generation.

Besides the flow pattern generation, the metachronal motion we created here can also be used for particle transportation. Preliminary results show that the natural sand particles can be transported against gravity by changing the rotation direction of the magnetic field, as we show in Supporting Information Movie S4.

## 3 Conclusions

In this paper, we introduced a new way to generate metachronal motion of magnetic artificial cilia. The key idea was to attach an array with identical cilia on a curved surface and apply a uniform rotational magnetic field to actuate them. The curvature introduces angular differences between neighboring cilia relative to the magnetic field, thereby inducing phase differences that drive metachronal motion across the cilium array. In contrast to the existing methods, our approach generates metachronal motion without the need to control the magnetic or geometrical properties of individual cilia or to realize complex time-dependent magnetic fields. Furthermore, by controlling the surface curvature, the metachronal wave propagation direction can be reversed while the beating direction of the individual cilia is preserved, so that we obtain different metachronal behaviors analogous to symplectic and antiplectic metachrony found in nature; such control is not possible with most existing methods for generating metachronal artificial cilium motion. However, metachrony is induced because of the surface curvature, which leads to the angle difference between the cilium axes and the applied magnetic field. The main limitation of this method is, therefore, that a surface with particular curvature is needed and must be fabricated, and this may not be practical for all applications. Our flow characterization experiments indicate the possibility of inducing specific flow patterns using the curvature-induced metachronal cilium motion, which depends on the local curvature of the surface. These patterns range from the creation and transportation of smaller vortices superposed on a larger vortex rotating in the other direction to the generation of overall directional flow with reversed local flow spots traveling in the overall flow direction but without inducing net flow in low-Reynolds-number conditions, which is another limitation of the method. Even if there is no net flow, the potential for generating flow circulation may offer useful applications. For example, these effects may in future work be further developed for achieving microfluidic mixing or the generation of specific flow patterns by more intricate designs of the surface curvature, which may be interesting for application in microfluidic devices in the domains of lab-on-a-chip or organ-on-a-chip. Another potential application is to achieve controlled particle transportation in microfluidic devices. Finally, by attaching magnetic artificial cilia to microscopic objects with a specifically designed curvature, it may be possible to induce propulsion by applying a simple rotating uniform field.

## 4 Experimental Section/Methods

### 4.1 Fabrication of Magnetic Artificial Cilia and Curved Glass Surfaces

The magnetic artificial cilia are fabricated using a micromolding process illustrated in Figure 1A. The dimensions of the magnetic artificial cilia are 15 μm in diameter and 150 μm in height. We fabricate the cilium mold from a fused silica glass slide by femtosecond laser machining. The properties of the glass are changed locally during exposure to a scanning focused femtosecond laser beam, so that the etching rate in KOH is enhanced; hence, after exposure, the written features are removed in a KOH ultrasonic bath at 85 °C. We treat the surface of the obtained glass molds with silane before using it so that the cilium patch can be easily peeled off the mold. The magnetic artificial cilia are made from a mixture of SIBS (Kaneka, Japan) and paramagnetic carboxylic iron particles (99.5%, Sigma-Aldrich) in a mass ratio of 1:1. Using hot embossing, we press the mixture in the mold at 150 °C with a pressure of 0.4 tons. Subsequently, we remove the top layer of the mixture at 160 °C, at which the mixture is still relatively soft. A layer of pure SIBS is then pressed on top of the cilium structures using the same procedure as used for the mixture but now applying 0.8 tons of pressure. Then, we bake the molded structure on a hot plate at 160 °C with a magnet underneath to align the magnetic particles along the cilium length. Finally, we peel the magnetic artificial cilia off the mold while observing the process under a stereo microscope. Note: we apply some isopropyl alcohol between the SIBS layer and the mold surface to decrease the adhesion, which eases the demolding process.

The curved surface is made of fused silica by femtosecond laser machining, as shown in Figure 1B. Similar to the fabrication procedure of the cilium mold, we write a pattern on a fused silica glass slide with a femtosecond laser beam. The written patterns are etched away in a KOH ultrasonic bath at 85 °C. We wash the glass substructures in a deionized water ultrasonic bath at room temperature to remove any remaining KOH before further use. Finally, the cilium patch is glued to the curved glass surface. The integrated cilia and curved glass substructure are schematically shown in Figure 1C for the convex structure.

### 4.2 Actuation Setup

A home-built rotational uniform magnetic field is used to actuate the cilia, as schematically shown in Figure 2A(i). The actuation system comprises two permanent magnets (50 × 50 × 12.5 mm^3^, remnant flux density of 1.2 T, Q-50-50–12.5-N, Supermagnete, Germany) positioned at a 50 mm separation and held in place by a PMMA framework. The magnetic field produced by the parallel magnets is about 150 mT in the center. The magnets are driven by an electric motor, which can be controlled by an in-house program through commercial software (ESCON Studio). A glass holder is fabricated to accommodate the samples precisely within the center of the uniform magnetic field, and its positioning is adjustable using an external XYZ stage.

### 4.3 Characterization of the Magnetic Artificial Cilium Motion

To characterize the motion of the magnetic artificial cilia, we designed a sample holder that was made by 3D printing (Formlabs 3 SLA printer, VividWarbler, Formlabs), as illustrated in Figure 2A(ii). To clearly observe the MAC motion, we cut off a single column of cilia from the whole cilium patch and glued it on top of the curved glass surface. The integrated cilia and glass surface are positioned in the front of the chamber. The recess for positioning the cilium structure has a length of 12 mm, a width of 4 mm, and a depth of 2 mm. A transparent glass slide was glued in front of the 3D-printed chamber to create a transparent optical window to observe the cilium motion clearly. A high-speed camera (Phantom, USA) mounted on a stereo microscope (Olympus, SZ61) was used to capture the cilium motion immersed in water in a side view. The recording frequency was 1000 times the beating frequency of the cilia. We used ImageJ to analyze the videos.

### 4.4 Characterization of the Flow Pattern

We observed the flow pattern in a closed PDMS channel, as illustrated in Figure 2A(iii). The PDMS channel, having a width and height both of 5 mm, was made by casting PDMS into a 3D-printed mold. To observe the flow pattern clearly, we bonded a transparent glass slide in front of the PDMS channel after plasma treatment. Then, we glued the integrated cilium array patch and curved glass surface into the channel next to the transparent glass slide. The cilium array patch used in the flow experiments has 10 rows of cilia; the center-to-center distance between the rows is 450 μm. Therefore, the width of the cilium path (4.065 mm) is close to the width of the PDMS channel (5 mm). Subsequently, we filled the channel with liquid and fully closed the channel by applying a coverslip on top of the system. To ensure low-Reynolds-number conditions, we used pure glycerol in all experiments. To characterize the flow pattern, we added red-colored polystyrene tracer particles with a diameter of 20 μm (PS-Red-19.9, microParticles GmbH, Germany). We used a CMOS camera (DFK 33UX252) connected to a stereo microscope to capture the flow speed at 25, 30, and 20 frames per second (fps) for metachrony no. 1, metachrony no. 2, and combined metachrony, respectively. The optical system was focused in the center of the channel. The videos were converted to 8-bit by ImageJ first and then we used the PIVlab of MATLAB to analyze the video. The setting of the PIVlab was as follows: The cross-correlation optimization was carried out in the frequency domain through FFT; subpixel accuracy was achieved by 2 × 3-point Gaussian interpolation; the interrogation area was 64 pixels, and the step was 32 pixels, which was around 80 μm for the concave and convex cases, while it was around 130 μm for the combined concave and convex case. The magnitudes of the measured velocities, shown in Figures 5 and 6, are extremely low, which leads to relatively high inaccuracies in the determined velocities. For such low velocities, particularly the effect of Brownian motion on the obtained experimental results must be considered. Since the radius of the tracer particles is *r* = 10 μm, their diffusion constant is  m^2^/s, where *k* is the Boltzmann constant, *T* is the absolute temperature, and η is the fluid viscosity taken as η = 1412 mPa s (i.e., glycerol). On the time scale of the beating of the cilia, *t*_D_ = 1 to 0.2 s (corresponding to 1–5 Hz), and the average diffusion length is therefore  = 0.005–0.002 μm, corresponding to effective velocities of 0.005–0.01 μm/s. These values are significantly lower than the maximum velocities found in our experiments (e.g., 1–5 μm/s in Figures 5 and 6). To experimentally verify this conclusion, we carried out flow experiments in which the cilia were not actuated, so that the sole cause of tracer particle motion was due to Brownian motion (since the effect of gravity can be neglected at the time scales of our measurements). The results are shown in Figure S1. These results confirm the low velocity range, while the velocity direction is random. This indicates that, despite the relatively high inaccuracies of the flow measurements, our conclusions about the emerging flow patterns are justified.

## Data Availability

The Supporting Information is available from the Wiley Online Library or from the author.

## Supporting Information Available

Local flow pattern measured when the cilia are not actuated (PDF)
Metachrony no. 1 of magnetic artificial cilia resulted by the convex surface (AVI)
Metachrony no. 2 of magnetic artificial cilia resulted by the concave surface (AVI)
Combined metachrony no. 1 and no. 2 of magnetic artificial cilia (AVI)
Preliminary sand particle transportation experimental results of the metachronal motion (MP4)

## Supplementary Material

am4c06884_si_001.pdf

am4c06884_si_002.avi

am4c06884_si_003.avi

am4c06884_si_004.avi
