# Supplementary material for: Curved Surfaces Induce Metachronal Motion of Microscopic Magnetic Cilia
Source: ACS Appl Mater Interfaces. 2024 Jul 10;16(29):38733–43. doi: 10.1021/acsami.4c06884 (PMC11284747; doi:10.1021/acsami.4c06884)
Supplement: Supplementary file 1 — am4c06884_si_001.pdf [file am4c06884_si_001.pdf]

Supporting Information

Curved Surfaces Induce Metachronal Motion of Microscopic Magnetic Cilia

Zhiwei Cui[1,2], Tanveer ul Islam[1,2], Ye Wang[1,2], Jaap den Toonder[1,2]*

1. Microsystems, Department of Mechanical Engineering, Eindhoven University of Technology, 5612 AE, Eindhoven, The Netherlands.

2. Institute for Complex Molecular Systems (ICMS), Eindhoven University of Technology, 5612 AJ, Eindhoven, The Netherlands

*j.m.j.d.toonder@tue.nl

Supporting movies

Movie S1: Metachrony No.1 of magnetic artificial cilia resulted by convex surface.

Movie S2: Metachrony No.2 of magnetic artificial cilia resulted by concave surface.

Movie S3: Combined metachrony No.1 and No.2 of magnetic artificial cilia.

Movie S4: Preliminary sand particles transportation experimental results of the metachronal motion

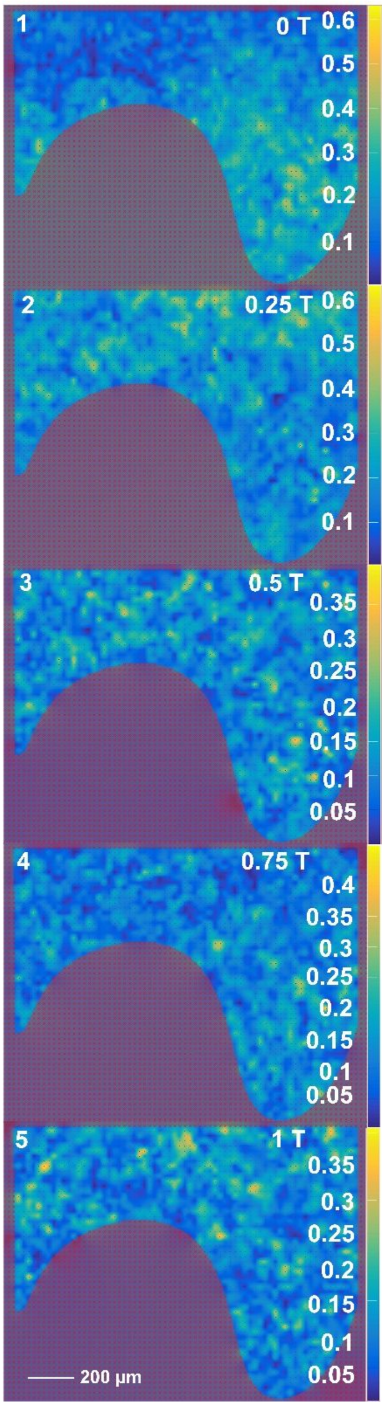

Figure S1: Local flow pattern measured when the cilia are not actuated, carried out to estimate the effect of Brownian motion on the flow results. Snapshots of the fluid velocity distribution are shown at 5 timepoints in one actuation cycle. The black arrows indicate the velocity direction, and the length of the arrow together with the color bars indicate the magnitude of the velocity. The unit of the color bars is µm/s.
